# Identification of lncRNAs involved in response to ionizing radiation in fibroblasts of long-term survivors of childhood cancer and cancer-free controls

**DOI:** 10.3389/fonc.2023.1158176

**Published:** 2023-04-27

**Authors:** Caine Lucas Grandt, Lara Kim Brackmann, Alicia Poplawski, Heike Schwarz, Federico Marini, Thomas Hankeln, Danuta Galetzka, Sebastian Zahnreich, Johanna Mirsch, Claudia Spix, Maria Blettner, Heinz Schmidberger, Manuela Marron

**Affiliations:** ^1^ Leibniz Institute for Prevention Research and Epidemiology – BIPS, Bremen, Germany; ^2^ Faculty of Human and Health Sciences, University of Bremen, Bremen, Germany; ^3^ Institute of Medical Biostatistics, Epidemiology and Informatics (IMBEI), University Medical Center of the Johannes Gutenberg University Mainz, Mainz, Germany; ^4^ Institute of Organismic and Molecular Evolution, Molecular Genetics and Genome Analysis, Johannes Gutenberg University Mainz, Mainz, Germany; ^5^ Department of Radiation Oncology and Radiation Therapy, University Medical Center of the Johannes Gutenberg University Mainz, Mainz, Germany; ^6^ Radiation Biology and DNA Repair, Technical University of Darmstadt, Darmstadt, Germany; ^7^ Division of Childhood Cancer Epidemiology, German Childhood Cancer Registry, Institute of Medical Biostatistics, Epidemiology and Informatics (IMBEI), University Medical Center of the Johannes Gutenberg University Mainz, Mainz, Germany

**Keywords:** weighted co-expression network analysis (WGCNA), differential gene expression analysis, RNA-Seq, radiation experiments, NGS - next generation sequencing, radiation response, KiKme Study

## Abstract

**Introduction:**

Long non-coding ribonucleic acids (lncRNAs) are involved in the cellular damage response following exposure to ionizing radiation as applied in radiotherapy. However, the role of lncRNAs in radiation response concerning intrinsic susceptibility to late effects of radiation exposure has not been examined in general or in long-term survivors of childhood cancer with and without potentially radiotherapy-related second primary cancers, in particular.

**Methods:**

Primary skin fibroblasts (n=52 each) of long-term childhood cancer survivors with a first primary cancer only (N1), at least one second primary neoplasm (N2+), as well as tumor-free controls (N0) from the KiKme case-control study were matched by sex, age, and additionally by year of diagnosis and entity of the first primary cancer. Fibroblasts were exposed to 0.05 and 2 Gray (Gy) X-rays. Differentially expressed lncRNAs were identified with and without interaction terms for donor group and dose. Weighted co-expression networks of lncRNA and mRNA were constructed using *WGCNA*. Resulting gene sets (modules) were correlated to the radiation doses and analyzed for biological function.

**Results:**

After irradiation with 0.05Gy, few lncRNAs were differentially expressed (N0: *AC004801.4*; N1: *PCCA-DT*, *AF129075.3*, *LINC00691*, *AL158206.1*; N2+: *LINC02315*). In reaction to 2 Gy, the number of differentially expressed lncRNAs was higher (N0: 152, N1: 169, N2+: 146). After 2 Gy, *AL109976.1* and *AL158206.1* were prominently upregulated in all donor groups. The co-expression analysis identified two modules containing lncRNAs that were associated with 2 Gy (module1: 102 mRNAs and 4 lncRNAs: *AL158206.1*, *AL109976.1*, *AC092171.5*, *TYMSOS*, associated with *p53-mediated reaction to DNA damage*; module2: 390 mRNAs, 7 lncRNAs: *AC004943.2*, *AC012073.1*, *AC026401.3*, *AC092718.4*, *MIR31HG*, *STXBP5-AS1*, *TMPO-AS1*, associated with *cell cycle regulation*).

**Discussion:**

For the first time, we identified the lncRNAs *AL158206.1* and *AL109976.1* as involved in the radiation response in primary fibroblasts by differential expression analysis. The co-expression analysis revealed a role of these lncRNAs in the DNA damage response and cell cycle regulation post-IR. These transcripts may be targets in cancer therapy against radiosensitivity, as well as provide grounds for the identification of at-risk patients for immediate adverse reactions in healthy tissues. With this work we deliver a broad basis and new leads for the examination of lncRNAs in the radiation response.

## 1 Introduction

About 70% of the human genome is transcribed into ribonucleic acids (RNA) (1, 2), whereas only 2-3% are subsequently translated into proteins (1, 3, 4). The transcripts that are not coding for any proteins are called non-coding RNAs (ncRNAs) and can be divided into small (e.g., micro-RNAs (miRNAs) and small interfering RNAs, < 200 nucleotides in length) and long non-coding RNAs (lncRNAs, longer than 200 nucleotides (5)). Contrary to messenger RNAs (mRNAs), ncRNAs are direct effectors not necessitating prior translation into proteins (5). Recently, RNAs have been identified as important players in the cellular response to ionizing radiation (IR), particularly by affecting the deoxyribonucleic acid (DNA) damage response (6). Involvement has been documented for several lncRNAs in response to high doses of IR (HDIR, ≥ 2Gy) (7) as well as low doses of IR (LDIR, ≤ 0.5Gy, (8)). lncRNAs can directly act as part of the damage response by binding repair factors at the site of a radiation-induced double-strand break or indirectly by regulating the cell cycle, transcription and/or translation, as well as working of miRNAs through acting as miRNA sponges (6). Moreover, lncRNAs can influence chromatin organization and regulate gene expression (9). While the clinical application of IR procures a high benefit in radiology and radiotherapy, the involved health risks due to unavoidable exposure of healthy tissue is not to be underestimated. These include deterministic acute tissue toxicities and stochastic long-term effects associated with HDIR as well as LDIR (10). The latter include radiogenic tumors, which can occur as second primary malignancies after radiotherapy with latency periods of several years to decades in long-term survivors of cancer (11). In particular, long-term survivors of childhood cancer are at the highest risk for late sequelae of DNA-damaging tumor therapies, including the development of a second primary malignancy (12) In Germany, 8% of long-term survivors of a tumor before age 15 develop a second primary malignancy within 30 years after the first cancer diagnosis (13). HDIR applied to the tumor volume during radiotherapy as well as to normal tissue at its margins is an established risk factor for second primary malignancies (14, 15), but also LDIR is considered a risk factor for carcinogenic late effects of IR exposure (15, 16). LDIR occurs as out-of-field doses in radiotherapy, e.g., as peripheral leakage and scatter radiation (17), but on a much larger civilizational scale through radiological imaging procedures such as computed tomography showing a dramatic rise in application through recent decades (18). However, the basic molecular mechanisms and intrinsic susceptibility to various adverse effects of the medical use of IR, particularly the occurrence of second primary cancers, have not been unraveled yet. In this context, this also applies to the role of lncRNAs in the cellular response to IR. To date, lncRNA expression analyses have been performed in a very limited number of studies in peripheral blood lymphocytes after very high radiation doses [60 Gy (19)] or in human (cancer) cell lines (20–23), rarely investigating the response to LDIR (20, 21).

The present study is the first to investigate the expression of lncRNAs in primary fibroblasts from a large population-based nested case-control study comprising long-term survivors of childhood cancer without (N1) or with at least one second primary malignancy (N2+), and cancer-free controls (N0) after HDIR (2 Gy) and LDIR (0.05 Gy). In addition, a co-expression network analysis with protein-coding transcripts was performed to decipher novel gene signatures and functions of lncRNAs in the cellular radiation response.

## 2 Materials and methods

### 2.1 Study design and participants

To examine the functional network between hereditary dispositions for sporadic childhood cancer, subsequent iatrogenic second primary neoplasms, and the cellular reaction to IR, the KiKme study was established (24). For this purpose, 591 participants were recruited from 2013 to 2019 and included in this nested case-control study. To this date, a body of work has been published, outlining the overall design, including a detailed description of participants and the analysis plan (24), determination of the experimental conditions yielding the most differentially expressed genes and overall experimental design (25), as well as an in-depth functional analysis of the protein-coding transcripts (26). For the latter as well as the project at hand, 156 participants (52 cancer-free controls, 52 long-term survivors of childhood cancer without, and 52 with at least one second primary neoplasm) over 18 years of age were selected and grouped in triplets (one of each donor group), matching them by age at sampling and sex. The two long-term childhood cancer survivors per triplet were additionally matched by first neoplasm, as well as age at and year of diagnosis. The cancer-free controls were recruited from patients at the Department of Orthopedic Surgery at the University Medical Center Mainz that were subject to elective procedures not associated with cancer. The long-term cancer survivors were selected if the first primary neoplasm was among the three most common pediatric cancers (leukemia, lymphoma, or a tumor of the central nervous system). To ensure that the second primary neoplasm had a potential radiogenic origin, all included second primary neoplasm occurred at an anatomic site that may have been exposed during a potential radiotherapy of the first primary neoplasm. [e.g., thyroid carcinoma, breast cancer, skin carcinoma, malignant melanoma, leukemia, or ependymomas and choroid plexus tumors (27)].

### 2.2 Samples and experiments

For this project, primary skin fibroblasts were obtained from 156 donors selected among the participants of the KiKme study. These samples were collected as 3 mm punch biopsies from the inside of the cubital region among long-term childhood cancer survivors and from the scar region of the surgery among cancer-free controls. For exposure to IR, cells were cultured and synchronized in G1 by confluency. The exposure to 2 Gy was created with 140 kilovolt X-rays at a dose rate of 3.62 Gy per minute for 0.55 minutes, exposure to 0.05 Gy with 50 kilovolt X-rays at a dose rate of 0.34 Gy per minute for 0.15 minutes. Both experiments were done using the D3150 X-ray Therapy System (Gulmay Medical Ltd, Byfleet, UK). The exposure to 0 Gy (sham-irradiation) was achieved by keeping the cells in the radiation device control room. All experiments occurred at room temperature, processing the triplets together in order to avoid batch effects. RNA was then extracted 4 hours after the IR-exposure.

### 2.3 RNA-Sequencing and processing

After the RNA isolation, RNA integrity number was measured using an Agilent 2100 Bioanalyzer using an Agilent RNA 6000 pico and nano assay. RNA concentration was measured with Qubit 2 and Qubit 4 fluorometers (Invitrogen, Germany) using the RNA BR and HS assay kits. Samples with RIN values <7 were excluded from subsequent library preparation. For the lncRNA samples, ribosomal RNA (rRNA) depletion from total human RNA was carried out using the QIAseq FastSelect–rRNA HMR Kit (QIAGEN GmbH, Hilden, Germany) according to the FastSelect−rRNA protocol specific for NEBNext^®^ libraries. For library preparations, we used the NEBNext^®^ Ultra II Directional RNA Library Prep Kit (New England BioLabs^®^, Frankfurt am Main, Germany) according to the manufacturer’s recommendations. Different dual index adaptors were used for multiplexing samples in one sequencing run. Library concentrations and quality were measured using a Qubit double-strand DNA high-sensitivity kit and QIAxcel capillary electrophoresis system with QIAxcel ScreenGel software (QIAGEN GmbH, Hilden, Germany). For both, mRNA-sequencing (mRNA-Seq) and the lncRNA-sequencing (lncRNA-Seq), the libraries were processed on a HiSeq2500 instrument (Illumina, San Diego, California, USA) set to high-output mode (Nucleic Acids Core Facility, Faculty of Biology, University of Mainz). Reads were generated using *TruSeq Single Read Cluster Kit v3* (Illumina, San Diego, California, USA) and *TruSeq SBS Kit v3* (Illumina, San Diego, California, USA). Here, single-end reads had a length of 51 base pairs using single indices (8), and a length of 43 base pairs using dual indices (8/8), for mRNA- and lncRNA-Seq, respectively. Base calling was performed by *Real-Time Analysis* (Version 1.8.4) and the resulting data were converted into FASTQ format using *bcl2fastq* (Version 1.8.4, Illumina, San Diego, California, USA).

### 2.4 Processing of lncRNA- and mRNA-Seq data

For the detection of differentially expressed genes and subsequent functional analysis, the mRNA- and lncRNA-Seq data were processed first. Raw reads were separated from the adapter sequences using *Trimmomatic* (28). For the trimming, we used a quality threshold of three for the removal of bases. Moreover, reads with an average quality below 15 over the span of four bases were then trimmed. The resulting processed reads were aligned to the human reference genome (GRCh38) using *STAR* (29). The expression per gene was then computed as the number of aligned reads per gene, quantified using *featureCounts* (30). The data were then normalized using the *voom* method (31), and *DESeq2* (32) for the differential expression and the weighted gene co-expression network analysis (*WGCNA*), respectively.

### 2.5 Analysis for differential gene expression

**Figure 1:**
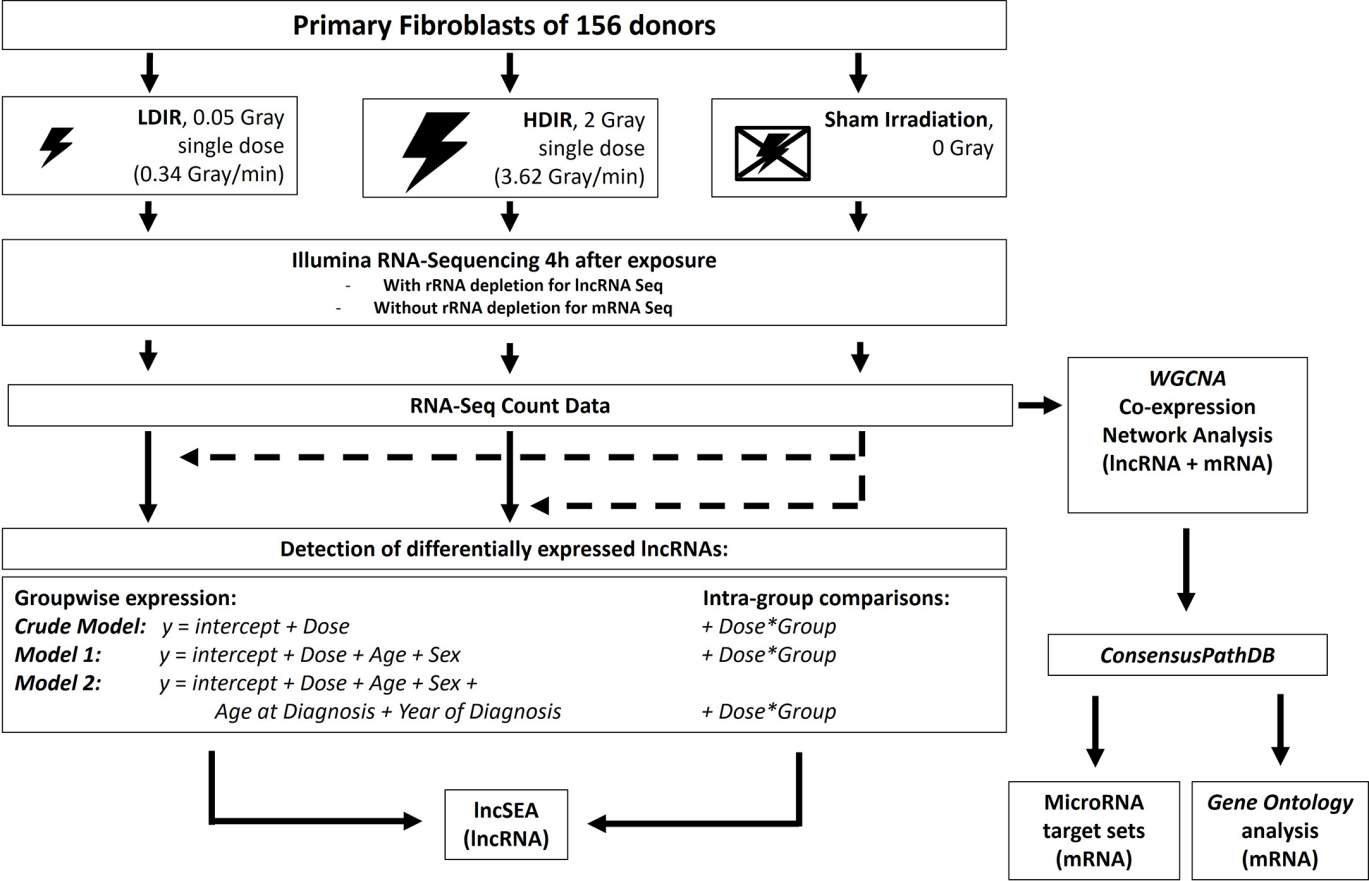
Experimental workflow: Primary fibroblasts of all donors were processed as matched triplets consisting of a donor with a first primary neoplasm only, a donor with a second primary neoplasm and a cancer-free control. The triplets were exposed to a low dose of ionizing radiation (LDIR, 0.05 Gray), a high dose of ionizing radiation (HDIR, 2 Gray), or were sham-irradiated (0 Gray). RNA was extracted 4h after exposure, sequenced on a HiSeq2500 *Illumina* device, and processed for the investigation of differentially expressed genes for each donor with regard to the regulation/level in sham-irradiated cells (dashed arrows). Differentially expressed lncRNAs as a result of groupwise-expression models, as well as data from the intra-group comparisons, were then subjected to the various analysis options of the *lncSEA2.0* platform. *DESeq2*-normalized data from mRNA- and lncRNA-Seq were additionally filtered for protein-coding and lncRNA transcripts, respectively, and then jointly used to detect co-expressed sets of mRNAs and lncRNAs with *WGCNA*. The resulting gene sets correlated with the radiation doses were then subjected to the *ConsensusPathDB* and analyzed for miRNA targets and Gene Ontology enrichment.

Differential gene expression of lncRNAs dependent on the radiation dose was computed with linear models implemented in the *limma* package (33). The individual donor was included as the block variable, additionally using *donor group* and *radiation dose* as factors of the model. The resulting log_2_ fold-changes (LFC) per gene and donor group after LDIR and HDIR were considered as the effect sizes on the individual transcriptome features, estimating the magnitude and direction of expression change between the respective groups (**Supplementary Tables S1A–D**, **Supplementary Figure S1**). For this purpose, we employed three different models: i.) considering the donor group (crude model), ii.) considering age and sex (model 1), and iii) considering age, sex, age at and year of diagnosis of the first neoplasm, and tumor type [(model 2), **Figure 1**]. Thus, we applied the specifications of model 2 to the subset of data including N1 and N2+ only. We additionally computed *p*-values for the interaction between the effect of the respective radiation dose and group to identify genes differentially expressed between the phenotype. Genes with a *p*-value adjusted at a false discovery rate [FDR, (34)] below 0.05 were reported as significant. The *p*-values from the separate analysis for each donor group have to be regarded as explorative since multiple pairwise comparisons were calculated. According to the best practice for reporting *p-*values originating from the R software, any *p*-values lower than 2.2x10^-16^ are reported as *p*-value<2.2x10^-16^ (35).

### 2.6 Processing of RNA-seq data

#### 2.6.1 Quality control

One sample (N2+ after HDIR) had to be removed due to a lack of appropriate quality. Thus, its samples and the samples from its matching group were not used for the identification of differentially expressed genes after HDIR. For the *WGCNA*, only the sample of concern was removed prior to network construction, as the donor group was later associated with a trait and not of relevance for the actual construction.

#### 2.6.2 Filtering of sequencing data

In order to merge the results of mRNA- and lncRNA-Seq, both datasets were filtered first. The mRNA data set was filtered for protein-coding genes, and the lncRNA-Seq data for lncRNAs, using the functional Ensembl GRCh38 annotations (36) retrieved *via BioMart v2.48.3* (37).

### 2.7 Weighted gene co-expression network

The *WGCNA* package allows for the examination of correlation patterns among a large set of genes. As result, it identifies clusters (called *modules*) of highly correlated transcripts. For the better legibility, these are then assigned colors that refer to each respective clustered group of co-expressed genes. All genes that were not clustered into a group of co-expressed transcripts were collected to the grey module. To construct robust weighted co-expression networks, we implemented the analysis with the *WGCNA* R package, following the instructions provided by its developers/creators. The mRNA- and lncRNA-Seq expression data were first merged and used to create a sample tree with the hclust function (method=“average”) in R (**Supplemental Figure 2**). Here, no further outliers were detected, and all 467 samples were used for the network construction. Next, we computed the powers (B) for a signed network to determine the power β_i_ used to establish an approximate scale-free topology. This was necessary to determine clusters of genes with high connectivity (the sum of connection strengths) and identify the few genes that were connected to many others (hub-genes), additionally making the network more robust in the process. In short, we examined the correlation between log_10_ of the connectivity (k) and the log_10_ proportion of genes with connectivity k (p(k)), where we regarded scale-free topology as approximately satisfied if r^2^ ≥ 0.9. Thus, we chose β_=_24. The network was then constructed using the blockwisemodules() function with corType=“bicor”, minModuleSize=15, reassignThreshold=0, and mergeCutHeight=0.20. The hub-genes were then calculated for selected modules using the intramodularConnectivity() function. Details for the described analyses can be followed and reproduced by the notebooks included in the repository https://github.com/clg1990/KiKme_lncRNA.

#### 2.7.1 Correlation of modules with traits

In order to correlate the colored modules with the available experimental metadata, each module was then assigned a single value that summarizes the expression direction, called *eigengene*. These eigengenes were then correlated to age, sex, donor group, and radiation dose, using the Pearson correlation coefficient. The belonging Student asymptotic *p*-values were computed for the correlation values and then adjusted at an FDR of 0.05.

### 2.8 Functional and interaction analyses of differentially expressed lncRNAs

Since lncRNAs can act as miRNA-sponges to suppress post-transcriptional regulation through miRNA-mRNA binding, we further examined prominently expressed lncRNAs for experimentally validated interactions with miRNAs stored in the *DIANA lncBASEv3* (38). Next, we identified coding transcripts competing for regulatory mechanisms of miRNAs and lncRNAs by further examining information on miRNAs identified and stored in the *lncBASEv3* for data on experimentally validated miRNA-mRNA-interactions in the *ENCORI* database (39). mRNAs associated with the previously retrieved miRNA-lncRNA data were finally integrated with the protein-coding genes present in the radiation modules from the *WGCNA*.

#### 2.8.1 Over-representation and Gene Ontology analysis of co-expressed mRNA

Ensemble Gene IDs from the co-expression modules associated with the radiation dose were used as gene set input for the Gene Ontology (GO) (40, 41) overrepresentation analysis, using the *ConsensusPathDB* (42). According to best practices, the genes from the modules were compared to a given list of total genes detected in the experiment, called background (**Supplementary Table S2A**). The resulting GO terms (**Supplementary Table S2B**) were then summarized with an allowed semantic similarity of 0.9 with the organism set to *Homo sapiens* using*REVIGO* (43), The results were then extracted as R script provided by the platform, translated into ggplot2 format for further modifications, and plotted as tree maps. In these, tile sizes of the tree maps were defined to represent the adjusted *p-*value of each respective GO term. Moreover, the genes from *WGCNA* modules associated with the radiation dose were examined for miRNAs (**Supplementary Table S2C** *, miRTarBase* v8.0 also provided by the *ConsensusPathDB*) known to regulate these transcripts, as well as joint transcription factors [**Supplementary Table S2D** (44)].

#### 2.8.2 Candidate list of lncRNAs of interest

To examine differentially expressed lncRNAs that were detected in our data with the results of other researchers, we created a list containing 70 lncRNAs of interest, known to be involved in the modulation of radio sensitivity (45), damage and repair in cancer cells, as well as those proposed as biomarkers for radiation damage (46) (**Supplementary Table S5**, **Supplementary Figure S6**). We then filtered our data for the genes in the candidate list and analyzed them with regard to their status as being differentially expressed across the donor groups and models.

## 3 Results

### 3.1 LDIR

#### 3.1.1 Differential expression of lncRNAs

**Figure 2:**
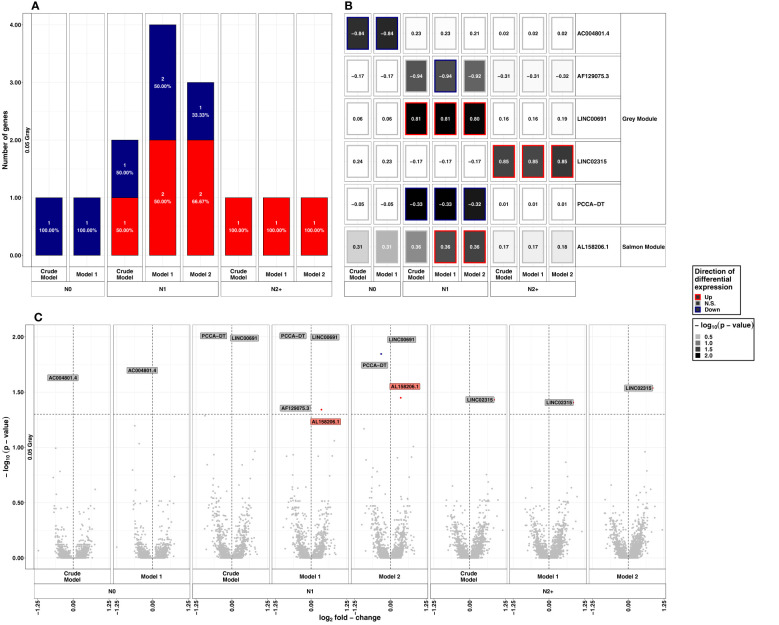
Summarized results on differential lncRNA expression after 0.05 Gray. Differentially expressed lncRNAs in irradiated compared to sham-irradiated fibroblasts from donors with a first primary neoplasm only (N1), donors with at least one second primary neoplasm (N2+), and cancer-free controls (N0) 4h after exposure to 0.05 Gray (false discovery rate adjusted *p*-value < 0.05). The data are presented for the crude model, model 1 (considering age at sampling and sex), and model 2 [considering age at sampling, sex, age at and year of diagnosis of the first neoplasm, and tumor type (not applicable for N0 data)]. In total 6225 lncRNAs were detected in the samples. Shown are **(A)** the proportion of up- (red) and downregulated (blue) genes stratified by radiation dose, group, and model, **(B)** a heat map of all differentially expressed lncRNAs, and **(C)** stratified by radiation dose, group, and model. The colors mentioned in the facets of **(B)** and used for the labels in **(C)** indicate the respective gene set the lncRNAs were assigned to (see **Figure 4**).

After exposure to LDIR, only 1-3 lncRNAs were differentially expressed across the donor groups and models, with no lncRNA being differentially expressed in more than one donor group (**Figure 2**). In N0, the only differentially expressed lncRNA was *AC004801.4* being detected as downregulated in all models (model 1: LFC=-0.84, *p-*value=0.02). In N1, *LINC00691* was up- (model 1: LFC=-0.80, *p-*value=0.01) and *Propionyl-CoA Carboxylase Subunit Alpha Divergent Transcript* (*PCCA-DT*) downregulated (model 1: LFC=-0.33, *p-*value=0.01) in all models after LDIR. Additionally, *AL158206.1* was upregulated in models 1 and 2 (model 1: LFC=0.36, *p-*value=0.04) and *AF129075.3* was downregulated in model 1 (LFC=-0.94, *p-*value=0.04). In N2+, *LINC02315* was upregulated in all models post-LDIR (model 1: LFC=0.85, *p*-value= 0.04). A detailed list of differentially expressed lncRNAs after LDIR is provided in **Supplementary Table S1C**. The additional analysis for interactions between the effect of radiation dose and the donor group did not identify lncRNAs with an adjusted *p*-value<0.05 in reaction to LDIR (**Supplementary Table S1D**). We thus applied a less conservative threshold of an unadjusted *p*-value<10^-5^. Application of this threshold resulted in the identification of three lncRNAs after LDIR. There were *AC004801.4* for N2/N1 versus N0 and N1 versus N0, *AC137932.3* for N2+ versus N0, and *AC073591.1*, for N2+ versus N1 post-LDIR (**Table 1** and **Supplementary Table S1E**).

**Table 1:** Overview of lncRNAs found for the analysis of interactions between the effect of radiation dose and donor group.

	Gene	Ensemble ID	N2+/N1 vs N0		N1 vs N0		N2+ vs N0		N2+ vs N1	
			unadjusted *p*-value	LFC	unadjusted *p*-value	LFC	unadjusted *p*-value	LFC	unadjusted *p*-value	LFC
0.05 Gray	AC004801.4	*ENSG00000257985*	3.10x10^-5^	0.96	6.10x10^-5^	1.07	–	–	–	–
	AC137932.3	*ENSG00000244577*	–	–	–	–	1.36x10^-5^	0.78	–	–
	AC073591.1	*ENSG00000257835*	–	–	–	–	–	–	1.65x10^-5^	-1.03
2 Gray	*AC069547.2*	*ENSG00000269165*	5.01x10^-6^	1.28	–	–	1.68x10^-5^	1.39	–	–
	*AC025284.1*	*ENSG00000260701*	4.24x10^-5^	0.84	–	–	–	–	–	–
	*LINC02139*	*ENSG00000278214*	6.61x10^-5^	0.87	–	–	–	–	–	–
	*LINC01099*	*ENSG00000251504*	–	–	8.76x10^-5^	1.39	–	–	–	–
	*AC073389.1*	*ENSG00000268584*	–	–	6.47x10^-5^	-0.68	–	–	–	–
	*AL162718.2*	*ENSG00000285694*	–	–	–	–	–	–	5.55x10^-5^	0.95

N2+=fibroblasts of donors with a first primary neoplasm in childhood and at least one second primary neoplasm,

N1=fibroblasts of donors with a first primary neoplasm in childhood, N0=fibroblasts of cancer-free controls;

LFC=log_2_ fold-change.

Genes were selected if their unadjusted p-value was below 10^-5^. Data adjusted for age at sampling and sex (model 1) are shown.

##### 3.1.1.1 Sensitivity analyses

After stratifying by sex, only *LINC00642* was downregulated in N0 males after LDIR (LFC=-1.34, *p*-value=5.84x10^-06^). Moreover, *LINC01909* (LFC=1.48, *p*-value=0.01), as well as *AL121772.1* (LFC=0.87, *p*-value=0.04) were upregulated in N1 males (**Supplementary Table S1E**, **Supplementary Figure S3A**). None of these lncRNAs were differentially expressed in the main analysis and *vice versa*. In no female donors (N0/N1/N2+), nor male N2+ there were any differentially expressed lncRNAs after LDIR. The removal of self-reported non-Caucasian participants (n=1) did not change the results of the differential expression analysis after LDIR (data not shown).

### 3.2 HDIR

#### 3.2.1 Differential expression of lncRNAs

**Figure 3:**
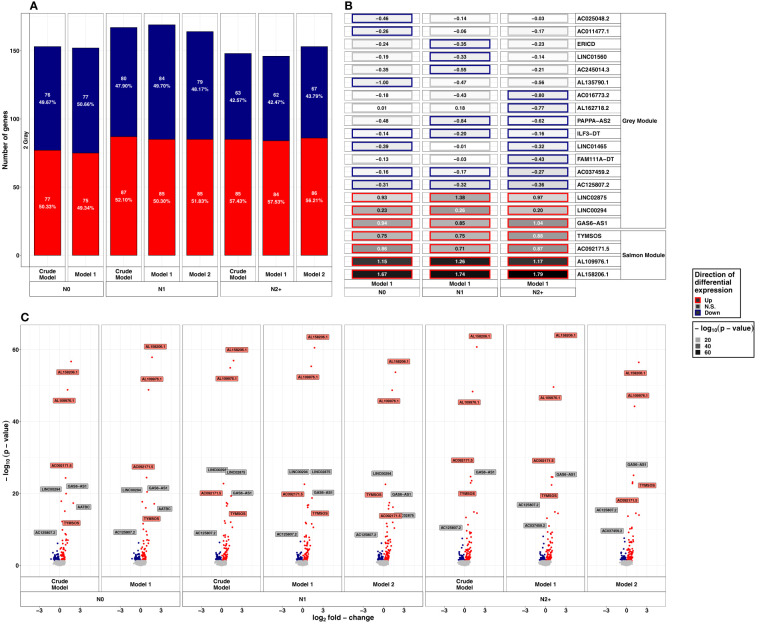
Summarized results on differential lncRNA expression after 2 Gray. Differentially expressed lncRNAs in irradiated compared to sham-irradiated fibroblasts from donors with a first primary neoplasm only (N1), donors with at least one second primary neoplasm (N2+), and cancer-free controls (N0) 4h after exposure to 2 Gray (false discovery rate adjusted *p*-value < 0.05). The data are presented for the crude model, model 1 (considering age at sampling and sex), and model 2 [considering age at sampling, sex, age at and year of diagnosis of the first neoplasm, and tumor type (not applicable for N0 data)]. In total 6225 lncRNAs were detected in the samples. Shown are **(A)** the proportion of up- and downregulated lncRNAs stratified by radiation dose, group, and model, **(B)** top 5 up- and downregulated lncRNAs with regard to the adjusted *p*-value of model 1, and, **(C)** volcano plots, stratified by radiation dose, group, and model. The colors mentioned in the facets of **(B)** and used for the labels in **(C)** indicate the respective gene set the lncRNAs were assigned to (see **Figure 4**).

After exposure to HDIR, the number of down- and upregulated lncRNAs was comparable in all donor groups and models with approximately 150 differentially expressed lncRNAs each (**Figure 3A** and **Supplementary Table S1C**). Among donor groups, the total number of differentially expressed lncRNAs was highest for N1 in all models (n_crude model_: 167, n_model 1_: 169, n_model 2_:164). Upregulated lncRNAs after HDIR showed smaller *p*-values in the range of 10^-10^ to 10^-64^ compared to downregulated genes [smallest *p*-value>10^-10^, **Figure 3C**]. All of the highest ranking upregulated lncRNAs were found in all groups and models after HDIR (**Figure 3B**), while the top downregulated lncRNAs with regard to *p*-value differed across groups, some surpassing the threshold for significance (**Figure 3B**). The two downregulated lncRNAs sorted by *p*-value were present across all donor groups and models after HDIR. These were *AC037459.2* (model 1: LFC_N0_: -0.16, *p*-value_N0_: 4.41x10^-04^; LFC_N1_: -0.17, *p*-value _N1_: 2.48x10^-04^; LFC_N2+_: -0.27, *p*-value_N2+_: 8.58x10^-07^) and *AC125807.2* (model 1: LFC_N0_: - 0.31, *p*-value_N0_: 5.29x10^-07^; LFC_N1_: - 0.32, *p*-value_N1_: 2.28x10^-07^; LFC_N2+_: - 0.36, *p*-value _N2+_: 8.64x10^-09;^ **Figure 3C**). Two lncRNAs were upregulated most strikingly with *p*-values below 10^-45^ in all donor groups and models. These were *AL109976.1* (model 1: LFC_N0_: 1.15, *p*-value_N0_: <2.2x10^-16^; LFC_N1_: 1.26, *p*-value_N1_: <2.2x10^-16^; LFC_N2+_: 1.17, *p*-value_N2+_: <2.2x10^-16^) and *AL158206.1* (LFC_N0_: 1.67, *p*-value_N0_: <2.2x10^-16^; LFC_N1_: 1.74, *p*-value_N1_: <2.2x10^-16^; LFC_N2+_: 1.79, *p*-value_N2+_: <2.2x10^-16^). As with LDIR, the analysis accounting for the interaction of donor group and HDIR resulted in no differentially expressed lncRNAs (**Supplementary Table S1D**). We thus again filtered for lncRNAs of interest below the threshold of the adjusted *p*-value but with an unadjusted *p*-value<10^-5^. Using this filter, we identified six lncRNAs after HDIR (*AC069547.2*, *AC025284.1*, *LINC02139*, *LINC01099*, *AC073389.1*, and *AL162718.2*; **Table 1**).

##### 3.2.1.1 Sensitivity analyses

After stratifying by sex, the most prominent lncRNAs after HDIR that were among the top upregulated lncRNAs with regard to the *p-*value in the main analyses were also present in the sensitivity analysis (*AL158206.1*, *AL109976.1*, *TYMSOS*, *LINC00294*, *GAS6-AS1*, *AATBC*, *LINC02875*; **Supplementary Table S1D**, **Supplementary Figure S2B**). The total number of differentially expressed lncRNA was halved compared to the number of the main analysis (N0_Female_: 68, N0_Male_: 66, N1_Female_: 63, N1_Male_: 59, N2+_Female_: 54, N2+_Male_: 78). Concerning downregulated lncRNAs, only *AC125807.*2 was downregulated in all male and female participants of all donor groups after HDIR. As with LDIR, the prior removal of self-reported non-Caucasian participants (n=1) did not change the results of the differential expression main analysis for exposure to HDIR (data not shown).

### 3.3 Weighted co-expression analysis of lncRNA and mRNA

**Figure 4:**
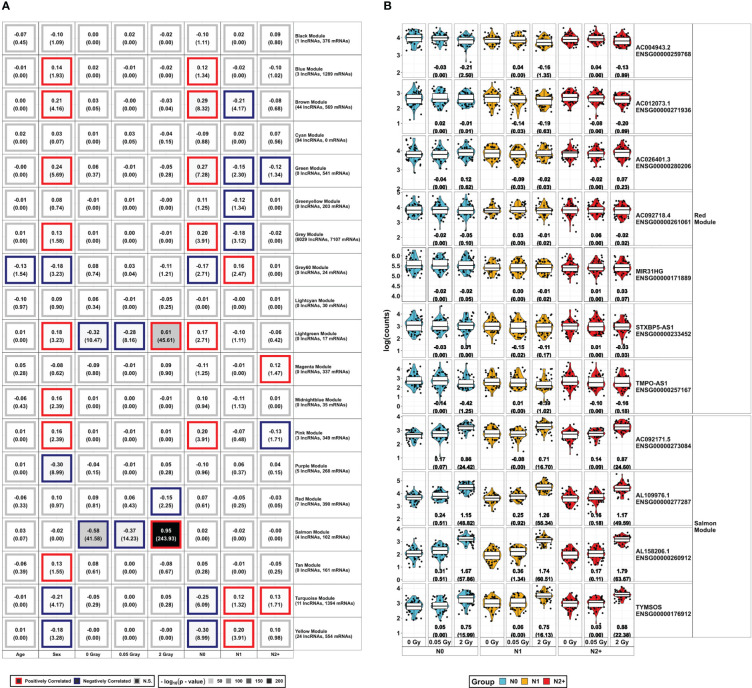
Overview of *WGCNA* Modules and lncRNAs found in modules associated with the radiation doses: **(A)** *DESeq2*-normalized protein-coding data from mRNA-Sequencing and lncRNAs from lncRNA-Sequencing were combined to construct a weighted co-expression network using *WGCNA*. The resulting gene sets (*Modules*) were assigned colors for better legibility. The respective expression of the lncRNAs in the radiation-associated modules is depicted in **(B)**, showing log_2_ fold-change and the false discovery rate adjusted *p*-value in brackets below for all combinations of radiation dose and donor group [fibroblasts from donors with a first primary neoplasm only (N1), donors with at least one second primary neoplasm (N2+), and cancer-free controls (N0)]. These data are taken from the model 1 of the differential expression analysis, accounting for sex and age at sampling.

Using *WGCNA*, we identified three modules that were significantly associated with HDIR (**Figure 4A**) and further examined the corresponding transcripts with regard to each modules’ intra-connectivity and the transcripts with the highest adjacency to the present lncRNAs. The visualization of the most important network features can be found in **Supplementary Figure S3**.

The salmon module was positively and strongly correlated with HDIR (r²=0.95, *p*-value<2.2x10^-16^) and negatively correlated to 0 Gray (r²=-0.58, *p*-value<2.2x10^-16^) and LDIR (r²=-0.37, *p*-value=10^-14^). Besides 102 coding genes, the salmon module encompassed four lncRNAs. These were *AL109976.1*, *AL158206.1*, *TYMSOS*, and *AC092171.5*, which were upregulated in differential expression analysis in all donor groups and models in response to HDIR (**Figures 3**, **4B**). *AL158206.1* was also upregulated by LDIR in N1 for models 1 and 2 (**Figure 2**). The five genes with the highest intra-modular connectivity were *MDM2*, *SESN1*, *CDKN1A*, *PPM1D*, and *BTG2* (**Supplementary Figure S2A**). With regard to adjacency, i.) *HSPA4L*, *BBC3*, *MDM2*, *IER5*, and *SESN1;* ii.) *CDKN1A*, *MDM2*, *TIGAR*, *BTG2*, and *HSPA4L;* iii.) *DDB2*, *BBC3*, *FDXR*, *IER5*, and *MRPL49;* iv.) *BLOC1S2*, *TIGAR*, *PPM1D*, *HSPA4L*, and *BTG2* were the top five genes to *AL109976*, *AL158206.1*, *TYMSOS*, and *AC092171.5* respectively (**Supplementary Tables S3B-E**).

The red module was only correlated with HDIR (r²=-0.15, *p*-value=10^-2^). Besides 390 coding mRNAs, there were seven lncRNAs present in this module (*AC004943.2*, *AC012073.1*, *AC026401.3*, *AC092718.4*, *MIR31HG*, *STXBP5-AS1*, and *TMPO-AS1*). Contrary to the salmon module, *AC004943.2* was the only differentially expressed lncRNA in the red module, downregulated only in N0 and N2+. The five genes with the highest intra-modular connectivity were *ANLN*, *PRC1*, *KIF11*, *CEP55*, and *TPX2* (**Supplementary Table S3F**). With regard to adjacency, i.) *KIF23*, *STIL*, *CENPO*, *DIAPH3*, and *MYBL1;* ii.) *TUBA1B*, *CCNA2*, *CENPO*, *STIL*, and *PRR11*; iii.) *PRR11*, *TOP2A*, *NCAPD2*, *KIF20B*, and *KNL1*; iv.) *CENPN*, *SHCBP1*, *DIAPH3*, *MYBL2*, and *CENPO;* v.) *ANP32E*, *DPYSL3*, *ITGB1*, *MYBL1*, and *TMPO;* vi.) *CKAP2*, *AURKA*, *ZWILCH*, *CDCA3*, and *PTTG1*; vii.) *CCNA2*, *CEP55*, *ANLN*, *KIF11*, and *KIF20B* were the top five genes to *AC004943.2*, *AC012073.1*, *AC026401.3*, *AC092718.4*, *MIR31HG*, *STXBP5-AS1*, and *TMPO-AS1*, respectively (**Supplementary Tables S3G-M**).

The lightgreen module contained 17 protein-coding genes and was correlated with the radiation doses [positively correlated to HDIR (r²=0.61, *p*-value<2.2x10^-16^), negatively correlated to 0 Gray (r²=-0.28, *p*-value=3.39x10^-11^), and LDIR (r²=-0.32, - *p*-value=6.92x10^-9^)]. In addition, it was positively associated with sex (r^2^ = 0.18, - *p*-value=5.89x10^-4^) and N0 (r^2^ = 0.17, *p*-value=1.95x10^-3^). The five genes with the highest intra-modular connectivity were *SUSD6*, *RIC1*, *TANC1*, *TCP11L1*, and *TNFRSF10D* (**Supplementary Table S3N**).

#### 3.3.1 Functional analysis of modules associated with exposure to radiation

In order to examine the co-expression modules for their functional implications, the protein-coding genes found in the salmon, red, and the lightgreen module were examined for the category *biological process* using the GO overrepresentation analysis in the *ConsensusPathDB* next (**Supplementary Table S2B**). Moreover, we additionally examined the mRNA of each module for the associated miRNA targets and transcription factors.

##### 3.3.1.1 Gene Ontology overrepresentation analysis

**Figure 5:**
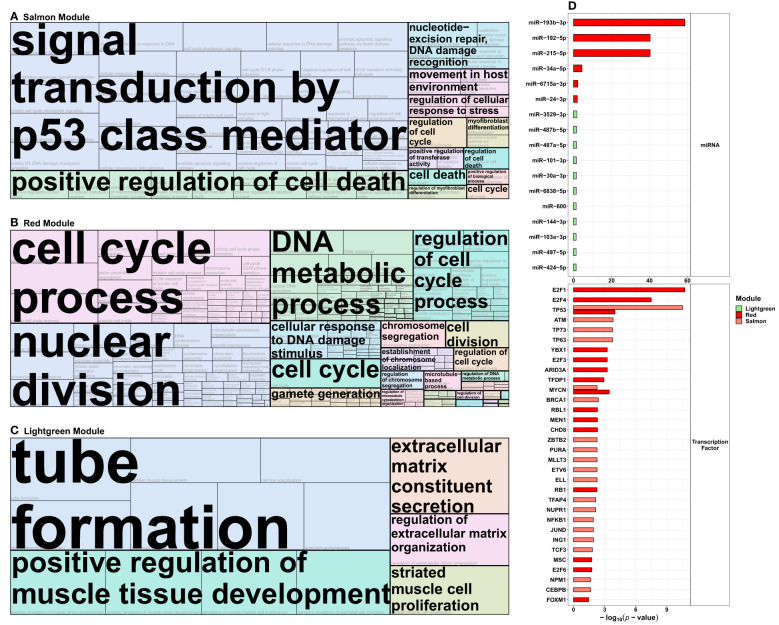
Functional analysis of the protein-coding transcripts from the radiation-modules identified in the co-expression network analysis. Protein-coding genes from the **(A)** salmon, **(B)** red, and **(C)** lightgreen (see **Figure 4**) *WGCNA* modules that were correlated with exposure to 2 Gray were used in Gene Ontology over-representation analysis *via ConsensusPathDB* and then summarized into tree maps *via* the *REVIGO* platform (allowed similarity 0.9, *Homo sapiens*, term category biological process). The term marked in large font indicates the representative term of each cluster of Gene Ontology terms, and the size of each tile represents the respective adjusted *p*-value. **(D)** depicts the miRNAs and transcription factors associated with the salmon, red, and lightgreen modules, respectively.

The respective data were used in *REVIGO* and clustered for the best term representatives. For the salmon module positively correlated with HDIR and negatively with LDIR and sham-irradiation, these representatives were *signal transduction by p53 class mediator* (*GO:0072331*), *positive regulation of cell death* (*GO:0010942*), *nucleotide-excision repair, DNA damage recognition* (*GO:0000715*), *regulation of cellular response to stress* (*GO:0080135*), and *cell death* (*GO:0008219*) among others (**Figure 5A**). For the salmon module, the sole *cellular component* was the *PCNA-p21-complex* (*GO:0070557*, **Supplementary Figure S4A**), among the main representatives for the *molecular functions* were *death receptor activity* (*GO:0005035*), *TRAIL binding* (*GO:0045569*), *damaged DNA binding* (*GO:0003684*), and *p53 binding* [(*GO:0002039*), **Supplementary Figure S4C**].

For the red module, only correlated with HDIR, the most prominent GO term-clusters concerning the *p*-value were various terms including the cell cycle and division [e.g., *cell cycle process* (*GO:0022402*), *nuclear division* (*GO:0000280*), r*egulation of cell cycle process* (*GO:0051726*)], *DNA metabolic process* (*GO:0055132*), c*ellular response to DNA damage stimulus* [(*GO:0034984*), **Figure 5B**]. For the *cellular components* or the *molecular functions*, *chromosomal region* (*GO:0098687*) and *DNA replication preinitiation complex* [(*GO:0031261*), **Supplementary Figure S4B**], *single stranded DNA binding* (*GO:0003677*) and *catalytic activity, acting on DNA* [(*GO:0140097*), **Supplementary Figure S4D**]. were among the main representatives, respectively.

For the lightgreen module, positively correlated with HDIR, sex, N0, as well as negatively correlated with LDIR and sham-irradiation, the most prominent GO term-clusters concerning *p*-value were *tube formation* (*GO:0035148*), *positive regulation of muscle tissue development* (*GO:1901863*), *extracellular matrix constituent secretion* (*GO:0070278*), *regulation of extracellular matrix organization* (*GO:19030539*), and *striated muscle cell proliferation* [(*GO:0014855*), **Figure 5C**]. No *cellular components* were identified for the lightgreen module, the sole representative for *molecular function* was *transmembrane signaling receptor activity* [(*GO:0004888*), **Supplementary Figure S4E**].

##### 3.3.1.2 miRNA targets and transcription factors

We further used the *ConsensusPathDB* to extract information on miRNAs that are regulators of the protein-coding genes of the radiation-associated modules from the *miRTarBase v8.0* (**Figure 5D** and **Supplementary Table S2C**). For the salmon module, no miRNAs were present in the overrepresentation analysis. The miRNAs concerning the adjusted *p*-value for the red module were *miR-193b-3p* (-log_10_(*p*-value)=58.60), *miR-192-5p* (-log_10_(*p*-value)=40.34), *miR-215-5p* (-log_10_(*p*-value)=40.33), *miR-34a-5p* (-log_10_(*p*-value)=4.44), *miR-6715a-3p* (-log_10_(*p*-value)=2.24), and *miR-24-3p* (-log_10_(*p*-value)=2.00). For the lightgreen module the top 5 miRNAs were *miR-3529-3p* (-log_10_(*p*-value)=1.76), *miR-487b-5p* (-log_10_(*p*-value)=1.57), *miR-487a-5p* (-log_10_(*p*-value)=1.57), *miR-101-3p* (-log_10_(*p*-value)=1.51), and *miR-30a-3p* [(-log_10_(*p*-value)=1.46).

We also computed associated transcription factors from *TRRUST v2* for the radiation-responsive modules (**Supplementary Table 2D**). The top 5 transcription factors for the salmon module concerning adjusted *p*-value were *TP53* (-log_10_(*p*-value)= 10.52), *ATM* (-log_10_(*p*-value)=3.82), *TP63* (-log_10_(*p*-value)=3.77), *TP73* (-log_10_(*p*-value)=3.77), and *BRCA1* (-log_10_(*p*-value)=2.40); and *E2F1* (-log_10_(*p*-value)=10.74), *E2F4* (-log_10_(*p*-value)=7.50), *TP53* (-log_10_(*p*-value)=4.00), *MYCN* (-log_10_(*p*-value)=3.46), and *YBX1* (-log_10_(*p*-value)=3.26) for the red module. The analysis did not identify transcription factors for the lightgreen module, due to the small number (n=17) of transcripts in this module.

Our examination using the *Diana lncBASEv3* returned 90 miRNAs known to interact with any of the 11 lncRNAs present in the radiation modules, as well as the 3 lncRNAs that were among the top downregulated lncRNAs in all donor groups after HDIR (*ILF3-DT*, *AC037459.*2, and *AC125807.2*) in healthy tissues. For these three, there were 24, 12, and 3 interacting miRNAs, respectively. With regard to the radiation modules, we identified 45, 20, 5, and one miRNA with experimentally validated interactions in normal tissue for *AL158206.1*, *TYMSOS, AL109976.1*, and *AC092171.5* of the salmon module, respectively (**Supplementary Table S4A**). For the lncRNAs of red modules, the experimentally validated number of interacting miRNAs was: *STXBP5-AS1* (n=47), *TMPO-AS1* (n=14), *AC092718.4* (n=12), *AC004943.2* (n=10), *AC012073.1* (n=8), *MIR31HG* (n=4), and *AC026401.3* (n=1). Analysis for overlap identified *hsa-miR-221-3p* to interact with all lncRNAs from the salmon module (**Supplementary Table S4B**, **Supplementary Figure S5**). No miRNAs were found to interact with all lncRNAs of either the salmon or the red module.

Regarding the list with lncRNAs of interest, 39 of 70 lncRNAs were present in our data. Of these, only six were differentially expressed in any model and donor group combination after HDIR, none after LDIR, although especially *PARTICLE* has been proposed to be an important lncRNA in reaction to LDIR (O'Leary, Ovsepian et al., 2015). *PVT1*, *DINOL*, and *NORAD* were upregulated in all groups and models, *PAPPA-AS1* was upregulated in all models of N1, *LINC00963* was downregulated in all models of N0 and N1, and *NKILA* was only upregulated in the crude model of N0. All of these transcripts were assigned to the grey module (**Supplementary Tables S1C, S8A**, **Supplementary Figure S6**).

## 4 Discussion

In the present nested case-control study we examined the expression of lncRNA in primary skin fibroblasts of long-term survivors of childhood cancer without and with at least one subsequent second primary neoplasm, as well as of cancer-free controls after exposure to different doses of IR. We detected similar responses to a low and a high dose of ionizing radiation between all donor groups and identified the lncRNAs *AL109976.1* and *AL158206.1* to be strongly associated with HDIR among all donor groups for the first time.

### 4.1 Differentially expressed lncRNAs

The lncRNAs *AC037459.2*, *AC125807.2*, and *ILF3-DT* were downregulated across all models of all donor groups post-HDIR. *AC037459.2* was previously associated with intracellular signal transduction and likely associated with carcinogenesis (47). Moreover, *AC037459.2* might act jointly with *PPP3CC*, a neighboring gene, to be involved with osteosarcoma and further cancers by regulating apoptosis and the MAPK signaling pathway, with the need for further research, as the authors themselves stated (48). *AC125807.2* was identified to be part of a network of competing endogenous RNA networks, also including *TMPO-AS1* (red module; only correlated with HDIR), to regulate *FAM82B* expression in lung adenocarcinoma, leading to poor prognosis (49) and was overexpressed in cutaneous melanoma (50). Moreover, *AC125807.2* was presented as part of a list of 14 immune-related lncRNAs which were associated with clinical outcomes in melanoma patients (51) and discussed as potentially involved in ferroptosis (52). Ferroptosis regulates cell death *via* lipid peroxidization, thus posing as a mechanism for tumor suppression. Recent data suggests that an increase in ferroptosis improved overall and progression-free survival after radiotherapy in cancer patients (53). In our radiation-responsive modules, however, no GO term comprising ferroptosis was found. *ILF-DT* is associated with autophagy and of prognostic value for cervical cancer together with 9 other lncRNAs, among those being *AL109976.1* of the salmon module [positively correlated only with HDIR, as well as negatively with LDIR and sham-irradiation (54)].

Comparing our data with Ding and colleagues (55), none of the lncRNAs in their study (*ITPK1-AS1*, *LINC00467*, *MIR22HG*, *DTX2P1-UPK3BP1-PMS2P11*, *OR2A1-AS1, LINC00173, TPTEP1*, *TINCR, LINC00336*, *C10orf111*, *TRHDE-AS1*, *MIR7-3HG*, *LINC00852*, *COLCA1*, *C20orf197*, *TTTY14*, and *MEG3*) were differentially expressed after LDIR in our results. Only *MIR22HG* was downregulated in all donor groups after HDIR in our analysis. Additionally, *RPP38-DT* (In 2015 termed *C10orf111*) was upregulated only in N0 after HDIR in our results, in contrast to a downregulation reported by Ding et al. (55). This might be explained by the fact that we adjusted for multiple testing, while Ding and colleagues did not. They also examined human fibroblasts, but these were HSF42 cell lines and radiation doses were defined differently (LDIR as 2 cGy and HDIR as 4 Gy). Moreover, only six of the lncRNAs in our candidate list of 70 lncRNAs that were reported to be associated with the radiation response were differentially expressed in our analysis, underlining the strong variability and tissue specificity of lncRNAs (56, 57).

### 4.2 Co-expression analysis

None of the modules that contained lncRNAs were simultaneously correlated to any radiation dose and any donor group, which was in line with our results on differentially expressed lncRNA including interaction terms where we did not identify prominent differences between the three donor groups. However, we identified lncRNAs with a strong signal in the co-expression analysis in response to HDIR, which were additionally identified as upregulated in the differential expression analysis.

#### 4.2.1 The salmon module

This module was positively correlated with HDIR but negatively correlated with LDIR and sham-irradiation. Its most prominent function was a plethora of GO-terms associated with the *p53*-mediated radiation response.

##### 4.2.1.1 AL109976.1

*AL109976.1* was previously reported to be associated with immune functions and used in the prediction of cervical cancer survival (58), among autophagy-related lncRNAs with prognostic value (54), as well as the top 20 downregulated lncRNAs in squamous cell tongue carcinoma (59).

##### 4.2.1.2 AL158206.1

Upregulation of *AL158206.1* was shown to act as a tumor suppressor by inhibiting proliferation and invasion in gastric cancer cells (60). Our finding of *AL158206.1* being upregulated and strongly associated with the *p53* pathway is supported by work on HepG2 cells exposed to cisplatin, a commonly used DNA damaging chemotherapeutic drug. Wang and colleagues associated this lncRNA with a comparable number of co-expressed transcripts (n=57) and identified p53 signaling as strongly affected pathways. They showed that *CDKN1A*, *TP53I3*, and *PPM1D* were upregulated by *AL158206.1.* Contrasting these findings, another work examining lncRNAs involved in the epithelial-mesenchymal-transition, associated with metastasis, identified *AL158206.1* as one of four lncRNAs having the strongest negative impact on the survival of patients undergoing drug treatment of hepatocellular carcinoma (61). Interestingly, we observed upregulation of *AL158206.1* and thus induction of protective radiation response after LDIR only in N1. This observation may suggest that in these donors, compared to N0 and N2+, there is an increased protective function in N1 in the case of low-dose genotoxic exposures of normal tissue, which occur, e.g., in the course of radiotherapy. This crucial difference in a lower-threshold cellular radiation response could therefore also reduce the risk of developing therapy-associated secondary malignancies in the N1 donors compared to N2+ donors. Regarding clinical relevance, *AL158206.1* may pose as a potent biomarker for the identification of at-risk patients for long-term adverse reactions in healthy tissues.

##### 4.2.1.3 TYMSOS

In gastric cancer, *TYMSOS* drives proliferation and migration through the sponging of miR-4739 (62) and in non-small cell lung carcinoma through the *FOXM1*/*TYMSOS*/*miR-214-3p*-axis (63). In our data neither miRNA was present the common regulators of the protein-coding transcripts of the radiation-responsive modules, very well keeping in mind that we examined healthy tissues, these patterns might be cancer-specific. Additionally, *TYMSOS* was used in a prognostic model as one of thirteen lncRNAs related to M6A regulators, a certain type of mRNA modification, that is also of importance in cancer (64), as one of 18 immune-related lncRNAs for clinical prediction in head and neck squamous cell carcinoma due to its potential to modulate tumor microenvironment (65).

##### 4.2.1.4 AC125807.2

Similar to the potential involvement of *AC125807.2* in ferroptosis, *AC092171.5* was part of a ferroptosis signature, containing 10 lncRNAs that also showed involvement in immune pathways that were additionally associated with cancer, further implicating the success of immune- and chemotherapy in lung adenocarcinoma patients and part of another immune-related signature, comprising of nine lncRNAs, employed to predict overall survival in pancreatic cancer (66).

The role of these transcripts for the immune system and various cancers is in line with the functional involvement of the co-expressed protein-coding transcripts in this work. In particular, involvement in the p53 pathway and its associated functions such as cell death, response to stress, and recognition, as well as repair, of DNA damage are key drivers of carcinogenesis when not functioning properly (67), or too well in the case of cancerous tissue evading cell death through overly strong stress response pathways (68). However, current literature does not provide information on the expression of these transcripts in the radiation response of healthy tissues, which does not allow a more in-depth comparison to other works, but underlines the importance of our observations.

#### 4.2.2 The red module

The red module was correlated with HDIR and associated with various processes of the cell cycle, as well as response to DNA damage. Interestingly, the analysis for differential expression did not determine six of the seven lncRNAs in this module to be differentially expressed post-LDIR or -HDIR. However, this module showed the weakest correlation of the three radiation-responsive modules, potentially implicating a signal very close to the detection limits of this analysis. Only *AC004943.2* was downregulated in N0 and N1 and *TMPO-AS1* was borderline significantly downregulated in N0 after HDIR.

##### 4.2.2.1 AC004943.2

*AC004943.2* was identified as one of three lncRNAs to be associated with *BRCA1/2* that enabled prognosis prediction as well as prediction of response to chemotherapy in patients with ovarian cancer (69). Interestingly, *AC004943.2* has been reported as part of a lncRNA set that is related to cuproptosis in head and neck squamous cell cancer (70). Such as the aforementioned ferroptosis, cuproptosis, is a novel pathway for programmed cell death, whereas this pathway involves excess intracellular copper, instead of iron (71).

##### 4.2.2.2 TMPO-AS1

Due to its dysregulation in several malignancies, *TMPO-AS1* has been a prominent transcript over the past years (72). *TMPO-AS1* functions by binding *FUS* and recruiting *p300* to the promoter of *TMPO*, thus activating its transcription (73) and being positively correlated (74). *TMPO* has been shown to induce proliferation and inducing cell cycle arrest and apoptosis in glioblastoma (75). Thus, unsurprisingly, the knockdown of *TMPO-AS1* suppressed growth and increased apoptosis in thyroid cancer (76), glioma progression (77), and cell proliferation and motility in pancreatic carcinoma (72). Moreover, reduced expression of *TMPO-AS1* increased overall survival and impaired growth of esophageal squamous cell carcinoma tumors (73). The expression of *TMPO-AS1* itself may be activated by *E2F1* (78) as being part of a positive regulatory loop, which was identified in the promotion of bladder cancer (79). Nevertheless, in our data N0 showed the lowest downregulation of *E2F1* post-HDIR (LFC_N0_=-0.27, LFC_N1_=-0.39, LFC_N2+_=-0.31), while exhibiting the largest reduction in *TMPO-AS1* expression and the only to be borderline significant (see **Figure 4**). Potentially, there are further mediators at play, that are not reflected in these data.

#### 4.2.3 The lightgreen module

This module was the only identified module that was correlated with the radiation doses and with N0 as one of the donor groups. Nevertheless, no lncRNAs but 17 mRNAs were involved in this module. These were however not present among the top genes identified in a prior work that examined the differential mRNA expression including an interaction term for the donor group [see Table 2, (26)].

### 4.3 Interaction of lncRNAs with miRNAs and transcription factors

We have previously reported data from this donor collective showing that the transcription factors *E2F1* and *TP53* were involved in response to radiation, with *E2F1* being enriched for N1 and N2+ post-HDIR, and *TP53* being enriched post-LDIR and post-HDIR in all donor groups (26). Moreover, all of the further top transcription factors, such as *E2F4* (80), *ATM* (81), *TP63* and *TP73* (82), *MYCN* (83), *YBX1* (84), *E2F3* (85), and *BRCA1* (86) have been reported to be of relevance for many aspects of the radiation response. The associated lncRNAs, such as *Linc01013*, reported to affect DNA damage repair *via YBX1* in endothelial cells (87) were not differentially expressed in our data. Noteworthy, *hsa-miR-221-3p*, the miRNA that was found to be interacting with all lncRNAs observed in the salmon module is also the only miRNA that is curated in the GO term *DNA repair* (*GO:0006281*) and prominently involved in cancer pathways (88, 89). This transcript was, however, not found in the analysis for involved miRNAs that regulate the protein-coding transcripts of the radiation responsive modules in our results.

### 4.4 Strengths and limitations

This is the first work to use primary skin fibroblasts from a large sample of a unique cohort of long-term survivors of childhood cancer with and without second primary neoplasms. Nevertheless, these results need to be interpreted bearing in mind some restrictions. lncRNAs are underlying a highly time- and tissue-specific expression and are present in far lower amounts compared to mRNA (90). Moreover, the largely unexplored intra-cellular cross-talk of long and further non-coding transcripts, as well as inter-cellular cross-talk of the DNA damage response after IR, known as the bystander effect (91), was not considered in this project. However, the high number of participants enabled us to facilitate differential expression analysis, with the appropriate statistical power, which, especially with lncRNA is heavily reliant on this fact, providing first insight on the topic and a potent basis to draw further projects from. Especially the interactions with miRNA are promising to elaborate on for further works (90), since it has been established that lncRNA and miRNA expression are important modulators for the radio sensitization of cancer cells (92), the findings of this work may facilitate new projects. The reported lncRNAs and their newly identified role may make them future targets in cancer therapy against radiosensitivity (92, 93). As resistance to the effect of applied ionizing radiation largely explains failure of cancer treatment (e.g., recurrence, metastasis, and survival), the molecular understanding and subsequent clinical modulation of such resistance to radiotherapy is at the core of facilitating future improvements in cancer treatment (94). Importantly, future projects of us and other are advised to complement their work by adding classical biological labwork (e.g., PCR validation of select candidates and design of further knockout experiments) to additionally support the findings, as well as to expand the analyses on tumor samples instead of healthy tissues to further examine implications on further aspects such as survival and metastasis, as others have done in similar work (95, 96).

## 5 Conclusion

We are the first to identify a set of lncRNAs, *AL158206.1* and *AL109976.1* most prominently, to be functionally involved in the radiation response through differential expression analysis of large-scale standardized radiation experiments and additional co-expression network analysis with protein-coding transcripts. These transcripts may pose as potent molecular targets, on the one hand for radio sensitization of tumors and on the other hand as predictive biomarkers of the individual response and risk of adverse effects of normal tissue to medical radiation exposures. Our work illustrates the relevance of investigating a broader spectrum and dimensions of transcriptomic cross-talk by miRNAs and non-coding transcripts in the context of IR exposures. Based on our findings, future investigation of the involvement of noncoding molecular units in the cellular radiation response and its associated health consequences is highly warranted to increase the efficiency of radiotherapy in tumor treatment as well as radiation protection.

## Data availability statement

The individual level RNA-seq data presented in this article are not publicly available, as they contain sensitive patient data underlying data protection rules. As stated in the patient information and signed informed consent documents for the ISIBELa project, such data may only be analyzed by the study team. Due to the regulations to protect the participants’ data and to ensure that they remain pseudonymized, the datasets generated and analyzed in this study are only available upon reasonable request. Any request to access the datasets should be directed to vl-isibel@uni-mainz.de.

## Ethics statement

We certify that all applicable institutional and governmental regulations concerning the ethical use of human volunteers were followed during this research. Approval by the Ethics Committee of the Medical Association of Rhineland-Palatinate was obtained (no. 837.262.12 (8363-F) and no. 837.440.03 (4102); **Supplementary Material** (Documents 1-4). Study participants will not undergo any procedures unless they give consent for examinations, collection of samples, subsequent analysis, and storage of personal data and collected samples. Study subjects can consent to single components of the study while abstaining from others. The patients/participants provided their written informed consent to participate in this study.

## Author contributions

MM developed the KiKme study and its design as the principal investigator. MM conceptualized the research idea on gene expression at different time points after exposure to high and low doses of ionizing radiation, designed the experiments, and developed the hypothesis on the role of lncRNAs in radiation response. LB and MM implemented the KiKme study. LB, CS, and MM conducted the participant recruitment, which was organized and planned by LB and MM. Former childhood cancer patients were identified, matched, and contacted by the German Childhood Cancer Registry. Doctors responsible for sample drawing were trained and supervised by MM and HS. The method of fibroblast sampling was established by DG and HS. HSZ takes care of the project ´s biobank and controls for the quality of all biosamples. Cell culture and radiation exposure of primary fibroblasts were established by SZ and DG. LB and SZ were responsible for the pseudonymization of all biosamples. The analysis pipeline for the project was developed by MM and AP. Analysis data of biosamples was processed by AP and TH. CG and AP conducted the statistical analysis. The final analysis of data concerning content was done by CG. All figures were designed and programmed by CG. CG, FM, and MM prepared the initial manuscript. All authors revised the manuscript critically for important intellectual content, approved the final version, and agreed to be accountable for all aspects of the work in ensuring that questions related to the accuracy or integrity of any part of the work are appropriately investigated and resolved. All authors contributed to the article and approved the submitted version.

## Glossary

Abbreviation: Full
ANLN: Anillin Actin Binding Protein
ANP32E: Acidic Nuclear Phosphoprotein 32 Family Member E
ATM: ATM Serine/Threonine Kinase
BBC3: Bcl2 Binding Component 3
BRCA1: Brca1 DNA Repair Associated
BRCA2: Brca2 DNA Repair Associated
BTG2: BTG Anti-Proliferation Factor 2
CCNA2: Cyclin A2
CDCA3: Cell Division Cycle Associated 3
CDCA5: Cell Division Cycle Associated 5
CDKN1A: Cyclin Dependent Kinase Inhibitor 1a
CENPN: Centromere Protein N
CENPO: Centromere Protein O
CEP55: Centrosomal Protein 55
CKAP2: Cytoskeleton Associated Protein 2
COLCA1: Colorectal Cancer Associated 1
DEG: Differentially Expressed Gene
DIAPH3: Diaphanous Related Formin 3
DINOL: Damage Induced Long Noncoding Ribonucleic Acid
DNA: Deoxyribonucleic Acid
DPYSL3: Dihydropyrimidinase Like 3
DTX2P1-UPK3BP1-PMS2P11: Dtx2p1-Upk3bp1-Pms2p11 Readthrough, Transcribed Pseudogene
E2F1: E2f Transcription Factor 1
E2F3: E2f Transcription Factor 3
E2F4: E2f Transcription Factor 4
FAM82B: Family With Sequence Similarity 82, Member B
FANCD2: Fa Complementation Group D2
FDR: False Discovery Rate
FOXM1: Forkhead Box M1
FUS: Fus Ribonucleic Acid Binding Protein
GO: Gene Ontology
Gy: Gray
HDIR: High Dose of Ionizing Radiation
HSPA4L: Heat Shock Protein Family A (Hsp70) Member 4 Like
IER5: Immediate Early Response 5
ILF3-DT: Ilf3 Divergent Transcript
IR: Ionizing Radiation
ITGB1: Integrin Subunit Beta 1
ITPK1-AS1: Itpk1 Antisense Ribonucleic Acid 1
KIF11: Kinesin Family Member 11
KIF20B: Kinesin Family Member 20b
KIF23: Kinesin Family Member 23
KNL1: Kinetochore Scaffold 1
LDIR: Low Dose of Ionizing Radiation
LFC: Log2 Fold-Change
LINC00173: Long Intergenic Non-Protein Coding Ribonucleic Acid 173
LINC00336: Long Intergenic Non-Protein Coding Ribonucleic Acid 336
LINC00467: Long Intergenic Non-Protein Coding Ribonucleic Acid 467
LINC00852: Long Intergenic Non-Protein Coding Ribonucleic Acid 852
LINC00963: Long Intergenic Non-Protein Coding Ribonucleic Acid 963
lncRNA: Long Non-Coding Ribonucleic Acid
M6A: N6-Methyladenosine
MDM2: Mouse Double Minute Homolog 2 Proto-Oncogene
MEG3: Mate Ribonucleic Acidly Expressed 3
MIR22HG: Mir22 Host Gene
MIR31HG: Mir31 Host Gene
MIR7-3HG: Mir7-3 Host Gene
miRNA: Micro Ribonucleic Acid
mRNA: Messenger Ribonucleic Acid
MYBL1: Myb Proto-Oncogene Like 1
MYBL2: Myb Proto-Oncogene Like 2
MYCN: Mycn Proto-Oncogene, Bhlh Transcription Factor
N0: Fibroblasts of Cancer-Free Controls
N1: Fibroblasts of Donors with a First Primary Neoplasm in Childhood
N2+: Fibroblasts of Donors with a First Primary Neoplasm in Childhood and at least one Second Primary Neoplasm
NCAPD2: Non-Smc Condensin I Complex Subunit D2
ncRNA: Non-Coding Ribonucleic Acid
NORAD: Non-Coding Ribonucleic Acid Activated By DNA Damage
OR2A1-AS1: Or2a1 Antisense Ribonucleic Acid 1
p21: Cyclin-Dependent Kinase Inhibitor 1
PAPPA-AS1: Pappa Antisense Ribonucleic Acid 1
PARTICLE: Promoter Of MAT2A Antisense Radiation-Induced Circulating Long Non-Coding RNA
PCNA: Proliferating Cell Nuclear Antigen
PPM1D: Protein Phosphatase, Mg2+/Mn2+ Dependent 1d
PRC1: Protein Regulator Of Cytokinesis 1
PRR11: Proline Rich 11
PTTG1: Pttg1 Regulator Of Sister Chromatid Separation, Securin
PVT1: Pvt1 Oncogene
REVIGO: Reduce And Visualize Gene Ontology
RIC1: Ric1 Homolog, Rab6a Gef Complex Partner 1
RNA: Ribonucleic Acid
RPP38-DT: Rpp38 Divergent Transcript
rRNA: Ribosomal Ribonucleic Acid
SESN1: Sestrin 1
SHCBP1: Shc Binding And Spindle Associated 1
STIL: Stil Centriolar Assembly Protein
STXBP5-AS1: Stxbp5 Antisense Ribonucleic Acid 1
SUSD6: Sushi Domain Containing 6
TANC1: Tetratricopeptide Repeat, Ankyrin Repeat And Coiled-Coil Containing 1
TCP11L1: T-Complex 11 Like 1
TIGAR: Tp53 Induced Glycolysis Regulatory Phosphatase
TINCR: Tincr Ubiquitin Domain Containing
TMPO: Thymopoietin
TMPO-AS1: Tmpo Antisense Ribonucleic Acid 1
TNFRSF10D: Tnf Receptor Superfamily Member 10d
TOP2A: DNA Topoisomerase Ii Alpha
TP53: Tumor Protein P53
TP53I3: Tumor Protein P53 Inducible Protein 3
TP63: Tumor Protein P63
TP73: Tumor Protein P73
TPTEP1: Tpte Pseudogene 1
TPX2: Tpx2 Microtubule Nucleation Factor
TRAIL: TNF-related Apoptosis-Inducing Ligand
TRHDE-AS1: Trhde Antisense Ribonucleic Acid 1
TTTY14: Testis-Specific Transcript, Y-Linked 14
TUBA1B: Tubulin Alpha 1b
TYMSOS: Tyms Opposite Strand Ribonucleic Acid
WGCNA: Weighted Gene Co-Expression Network Analysis
YBX1: Y-Box Binding Protein 1
ZWILCH: Zwilch Kinetochore Protein
