# Supplementary material for: Identification of lncRNAs involved in response to ionizing radiation in fibroblasts of long-term survivors of childhood cancer and cancer-free controls
Source: Front Oncol. 2023 Apr 27;13:1158176. doi: 10.3389/fonc.2023.1158176 (PMC10174438; doi:10.3389/fonc.2023.1158176)
Supplement: Supplementary file 1 [file DataSheet_1.zip › Data Sheet 1/Figure S2A.DOCX]

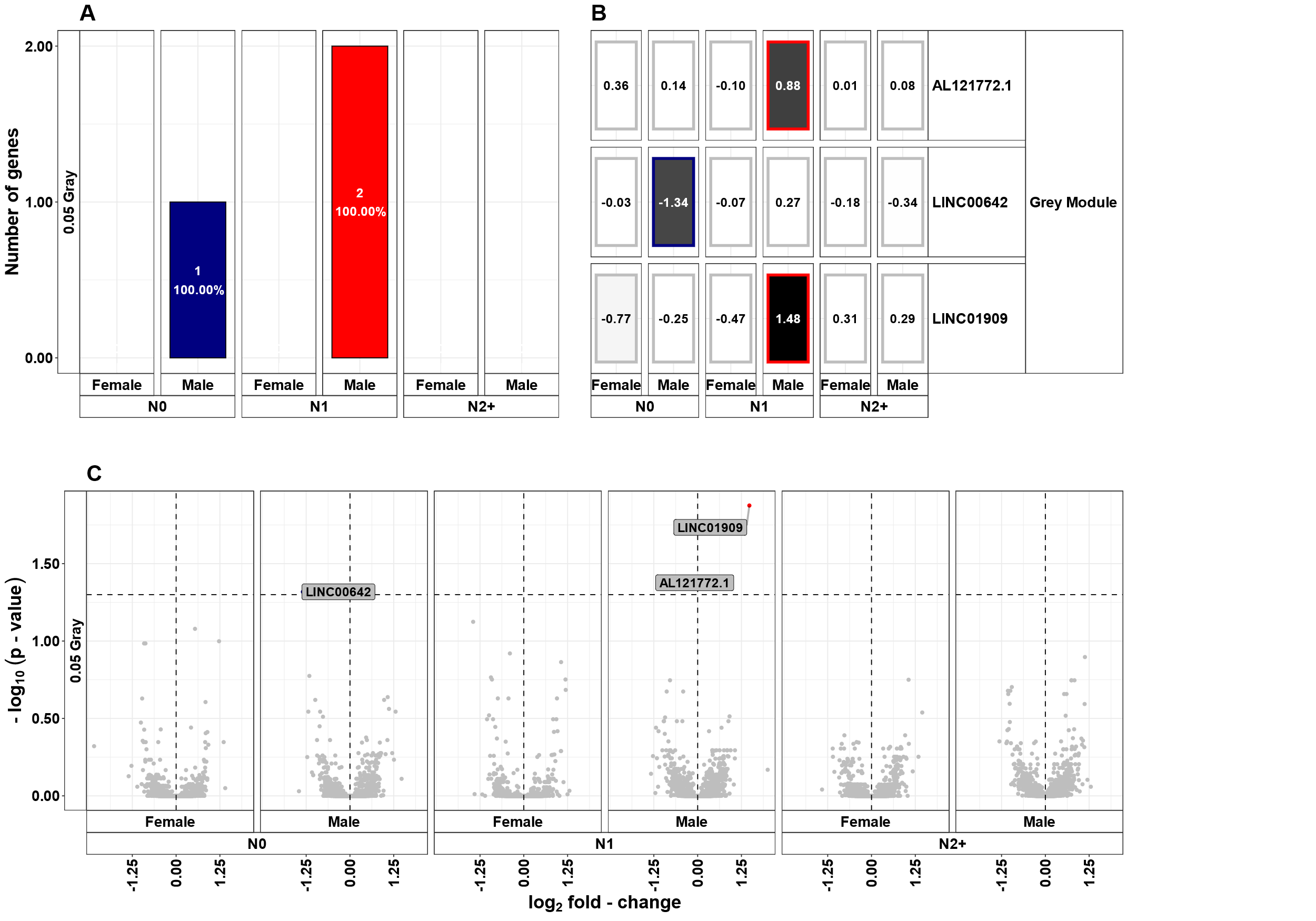


**Supplementary Figure S2A: Differential lncRNA expression, stratified by sex following 0.05 Gray (LDIR).** Differentially expressed lncRNAs in irradiated compared to sham-irradiated fibroblasts from donors with a first primary neoplasm only (N1), donors with at least one second primary neoplasm (N2+), and cancer-free controls (N0) 4h after exposure to 0.05 Gray (false discovery rate adjusted *p*-value < 0.05). The data are presented adjusted for age. In total 6225 lncRNAs were detected in the samples. Shown are A.) the proportion of up- (red) and downregulated (blue) genes stratified by radiation dose, group, and model, B.) a heat map of all differentially expressed lncRNAs, and C.) stratified by radiation dose, group, and model. The colors mentioned in the facets of B.) and used for the labels in C.) indicate the respective gene set the lncRNAs were assigned to (see Fig. 4).
