# Supplementary material for: Identification of lncRNAs involved in response to ionizing radiation in fibroblasts of long-term survivors of childhood cancer and cancer-free controls
Source: Front Oncol. 2023 Apr 27;13:1158176. doi: 10.3389/fonc.2023.1158176 (PMC10174438; doi:10.3389/fonc.2023.1158176)
Supplement: Supplementary file 1 [file DataSheet_1.zip › Data Sheet 1/Figure S5.DOCX]

**
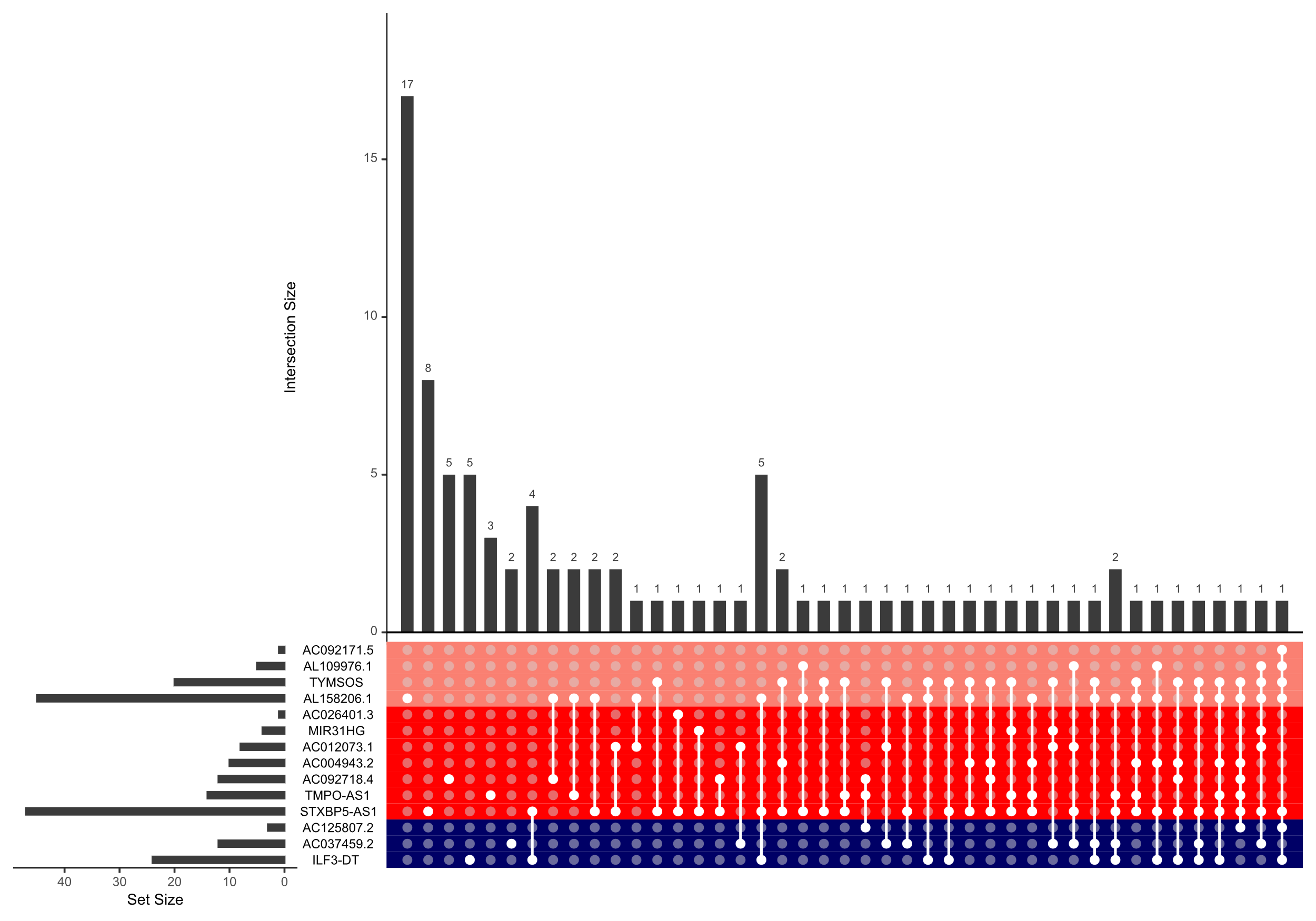
**

**Supplementary Figure S5: Visualization of the intersecting miRNAs from the miRNA target analysis per lncRNA of interest.**
