# Supplementary material for: Identification of lncRNAs involved in response to ionizing radiation in fibroblasts of long-term survivors of childhood cancer and cancer-free controls
Source: Front Oncol. 2023 Apr 27;13:1158176. doi: 10.3389/fonc.2023.1158176 (PMC10174438; doi:10.3389/fonc.2023.1158176)
Supplement: Supplementary file 1 [file DataSheet_1.zip › Data Sheet 1/Figure S6.DOCX]

**
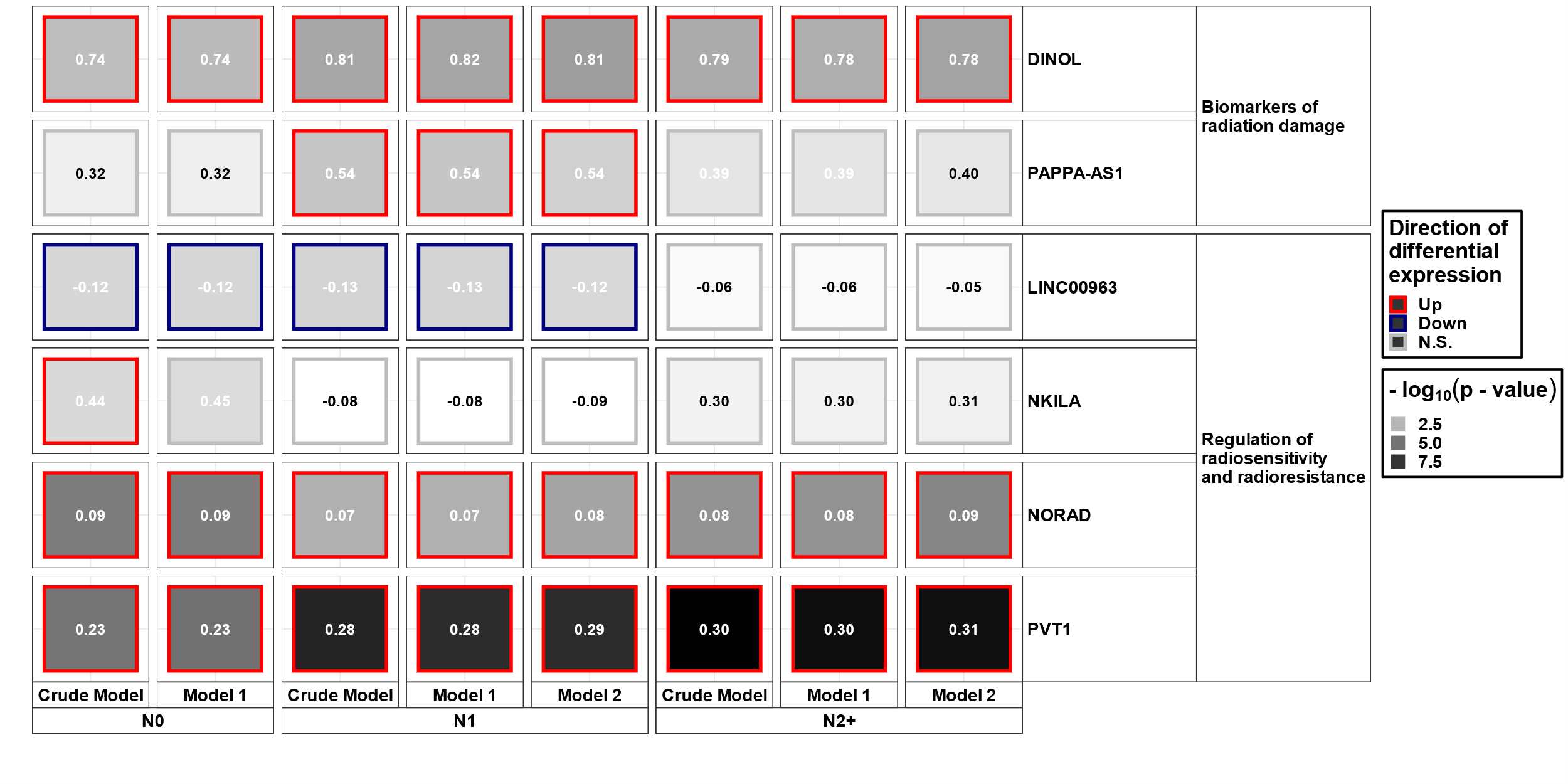
**

**Supplementary Figure S6: Visualisation of differential expression data of lncRNAs potentially involved in the radiation response according to the literature.** Differentially expressed lncRNAs in irradiated compared to sham-irradiated fibroblasts from donors with a first primary neoplasm only (N1), donors with at least one second primary neoplasm (N2+), and cancer-free controls (N0) 4h after exposure to 2 Gray (false discovery rate adjusted p-value < 0.05). The data are presented for the crude model, model 1 (considering age at sampling and sex), and model 2 [considering age at sampling, sex, age at and year of diagnosis of the first neoplasm, and tumor type (not applicable for N0 data)]. In total 6225 lncRNAs were detected in the samples. Shown is the heat map of differentially expressed lncRNAs that were also present in the literature review.
