# Supplementary figures and images for: Identification of lncRNAs involved in response to ionizing radiation in fibroblasts of long-term survivors of childhood cancer and cancer-free controls
Source: Front Oncol. 2023 Apr 27;13:1158176. doi: 10.3389/fonc.2023.1158176 (PMC10174438; doi:10.3389/fonc.2023.1158176)

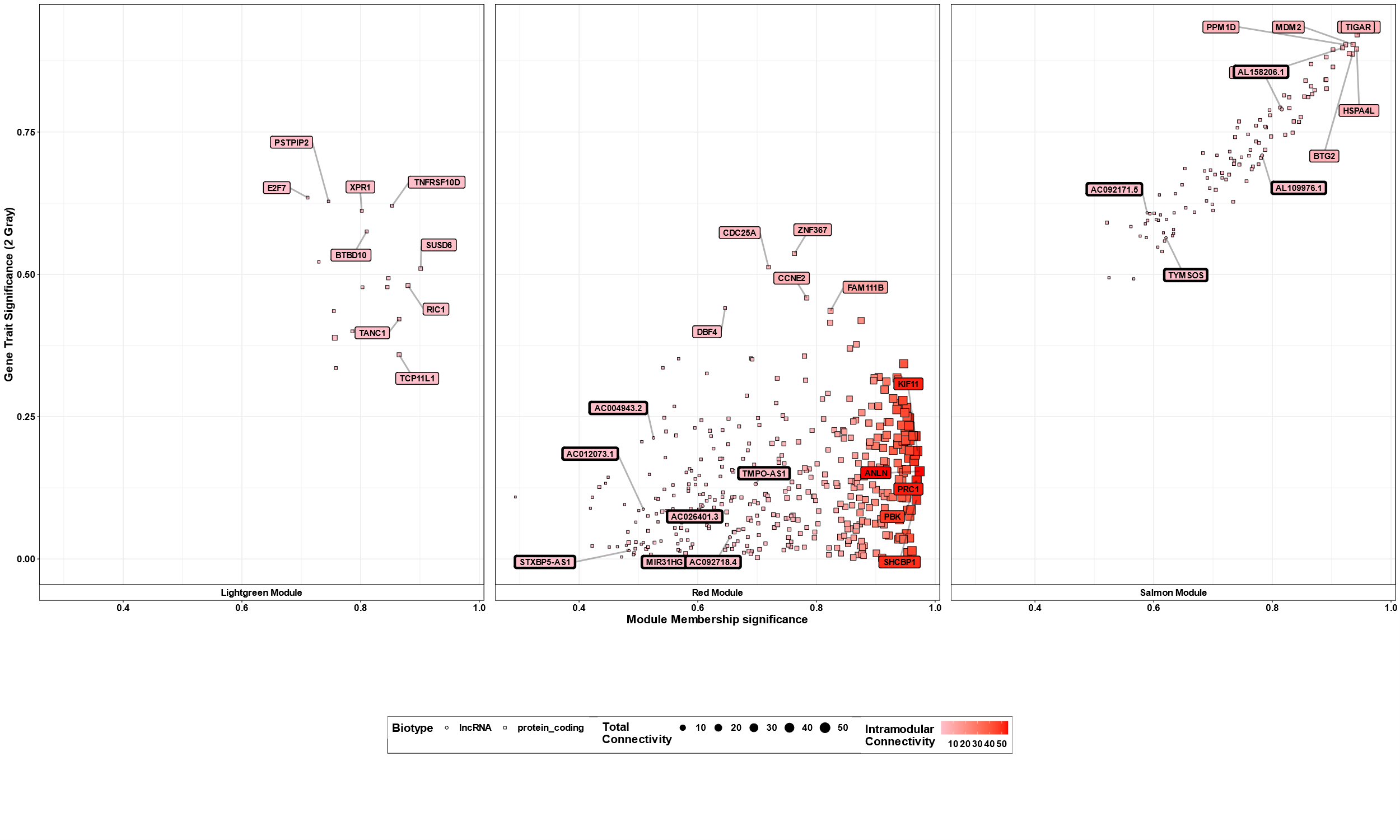


**Supplementary Figure S3: Further Network Analysis - Visualization of the network analysis data.**

Supplement: Supplementary file 1 [file DataSheet_1.zip › Data Sheet 1/Figure S3.DOCX]
